# Nesting innovations in neotropical parrots associated to anthropogenic environmental changes

**DOI:** 10.1002/ece3.10462

**Published:** 2023-09-01

**Authors:** Pedro Romero‐Vidal, Guillermo Blanco, Fernando Hiraldo, José A. Díaz‐Luque, Álvaro Luna, Daiana Lera, Sergio Zalba, Martina Carrete, José L. Tella

**Affiliations:** ^1^ Department of Physical, Chemical and Natural Systems Universidad Pablo de Olavide Sevilla Spain; ^2^ German Centre of Integrative Biodiversity Research (iDiv) Halle‐Jena‐Leipzig Leipzig Germany; ^3^ Department of Evolutionary Ecology Museo Nacional de Ciencias Naturales CSIC Madrid Spain; ^4^ Department of Conservation Biology Doñana Biological Station CSIC Sevilla Spain; ^5^ Endangered Conservation Consultancy Málaga Spain; ^6^ Department of Health Sciences, Faculty of Biomedical and Health Sciences Universidad Europea de Madrid Madrid Spain; ^7^ GEKKO, Grupo de Estudios en Conservación y Manejo, Departamento de Biología, Bioquímica y Farmacia Universidad Nacional del Sur Bahía Blanca Argentina

**Keywords:** breeding behavior, cavity nesters, nesting‐site availability, parrots, nesting‐site plasticity

## Abstract

Parrots are among the most diverse and widely distributed groups of birds and one of the most threatened bird orders mainly due to habitat loss and illegal poaching. Most parrots are obligate cavity nesters, so the logging of mature trees and the transformation of natural cliffs represent important threats to their conservation. Here, we report novel observations of Neotropical parrots nesting in previously unrecorded substrates. We show the first documented case of the cliff‐nesting burrowing parrots trying to breed at ground level in an abandoned burrowing owl cavity. Additionally, we provide the first documented observations of this species attempting to nest in building cavities in three urbanized areas of Argentina. Moreover, we report data from four countries of 148 pairs of eight species typically breeding in tree cavity using palm tree bracts as nest sites. Behavioral plasticity in nest sites may allow parrots to maximize their nesting success by exploiting alternative breeding substrates. Moreover, these novelties could contribute to cope with habitat loss and further transformation. However, further research is needed to assess the consequences of these nesting innovations in terms of individual fitness and population dynamics as well as potential factors promoting their appearance.

## Graphical abstract

Most parrots are obligate cavity nesters, so the logging of mature trees and the transformation of natural cliffs represent important threats to their conservation. Here, we report novel observations of Neotropical parrots nesting in previously unrecorded substrates. These novelties could contribute to cope with habitat loss and further habitat transformation.

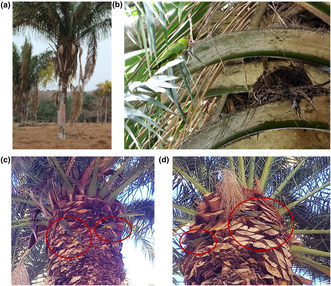

## 1 INTRODUCTION

Nest selection constitutes a key factor in the reproductive success of all bird species, conditioning habitat selection and even determining their natural history (Brightsmith, 2005a; Clark & Shutler, 1999; Martin, 1998; White Jr et al., 2006). Birds show great variability in their nesting strategies, and different groups can be established depending on the ability of individuals to build their own nests (vs. those that rely on existing substrates for nesting; Burns, 1924), or the substrate used (open vs. cavity nesters; Martin & Li, 1992). More specific classifications can be added. For example, cavity nesters—species that need holes to breed—can be divided into primary excavators, which dig their own cavities in trees or ravines, and secondary cavity nesters, which rely on existing cavities (Cockle et al., 2011). Secondary cavity nesters may thus be constrained by the number of available breeding sites and the abundance of primary cavity nesters that can provide nesting resources (Robles & Martin, 2014). Secondary cavity nesters can be therefore exposed to increased competition for the limited nesting sites, and increased risk of parasitism and predation (Brightsmith, 2005a, 2005b).

Parrots (Psittaciformes) constitute an example of secondary cavity nesters (Parr & Juniper, 2010; Renton et al., 2015), with exceptions such as: (i) the monk parakeet (*Myiopsitta monachus*), which digs its own stick nests in a variety of natural and artificial substrates (Hernández‐Brito, Martina Carrete, et al., 2021); (ii) the burrowing parrot (*Cyanoliseus patagonus*), which dig its own cavities in cliffs and ravines (Masello & Quillfeldt, 2012); and (iii) species from the genera *Eupsittula* and *Brotogeris*, which can excavate cavities in termite mounds (Forshaw, 2010). The distribution and abundance of parrots are influenced by the availability of suitable cliffs and tree holes for nesting (Parr & Juniper, 2010). In fact, about one third of the c. 400 extant parrot species are threatened due to habitat loss and degradation (Berkunsky et al., 2017; Olah et al., 2016, 2018) that reduce the availability of breeding cavities. This shortage not only has a direct impact by limiting the breeding population size of parrots through density‐dependent processes, but also by increasing, for example, nest predation due to a lack of good quality cavities (Fisher & Wiebe, 2006). However, in these resource scarcity scenarios, some individuals may make innovations (Lee & Moura, 2015; Morand‐Ferron et al., 2011; Reader & Laland, 2003) that may fix in populations if they have fitness advantages.

Here we provide evidence of parrots using previously unrecorded nesting sites, possibly in response to limited or scarce traditional nesting substrates. Our observations correspond to a wide variety of Neotropical species, living in different countries, habitats, and biomes. These observations may contribute to advance our understanding of the adaptive mechanisms and processes that trigger behavioral innovations.

## 2 METHODS

**FIGURE 1:**
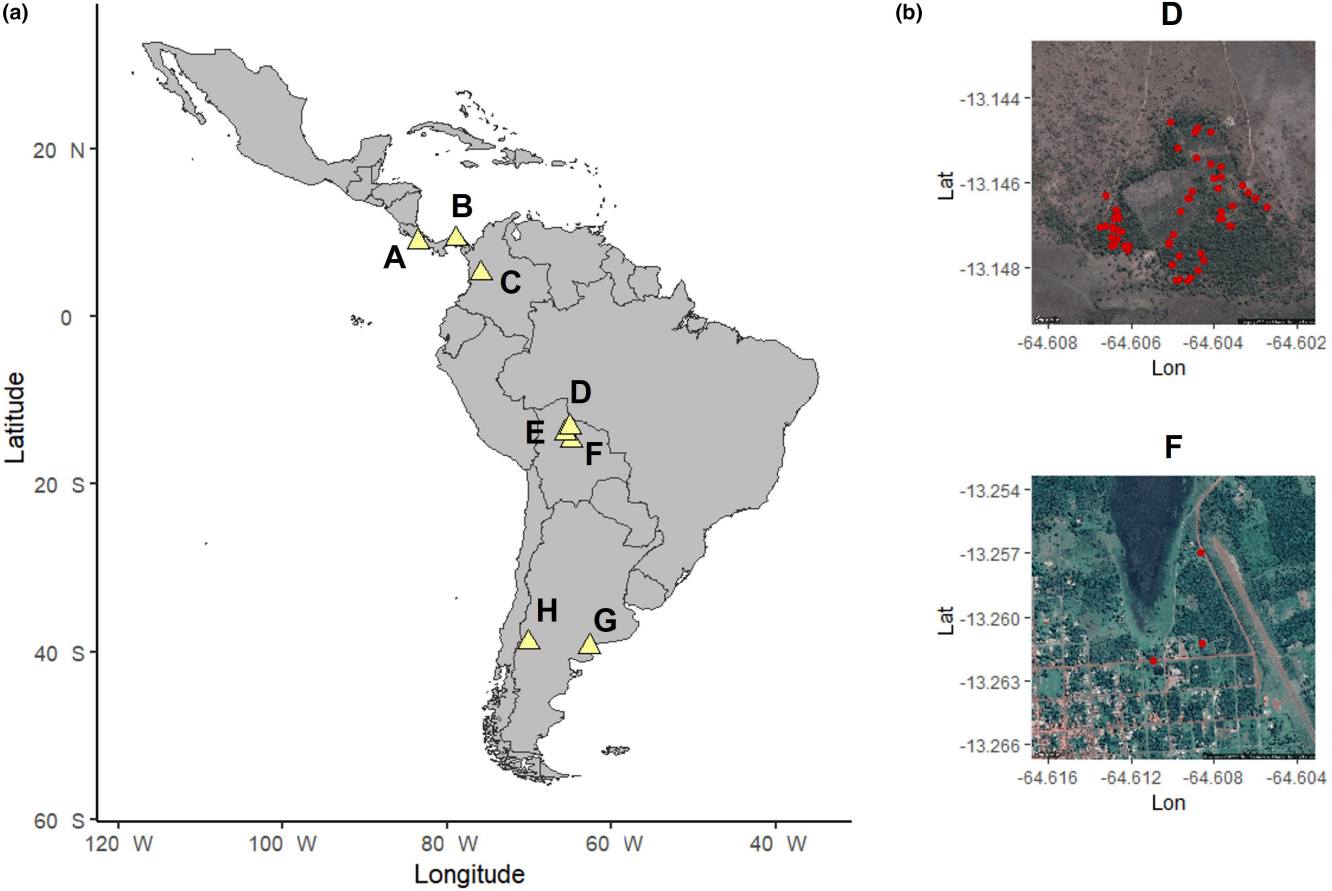
(a) Map showing the different locations where observations were performed (A–H). (b) Example of the nest distribution in palm tree bracts in two of the Bolivian sites (D and F).

From 2002 to 2019, we conducted a long‐term monitoring of rural and urban burrowing owls (*Athene cunicularia*; e.g., Mueller et al., 2018; Luna et al., 2021; Figure 1) and burrowing parrots (*Cyanoliseus patagonus*; Tella et al., 2014; Lera et al., 2022) (Figure 1) in Bahía Blanca and nearby areas (Argentina), where we casually observed several previously undocumented nesting events of burrowing parrots. During these monitoring, all breeding areas suitable or previously used by the species were repeatedly visited annually to assess occupation and breeding success. Burrowing parrots may breed solitarily, although they usually form colonies ranging from a few pairs to many thousands. Individuals excavate their own burrows by tunneling into sandstone, limestone, and earth cliffs (Masello et al., 2001, 2006; Masello & Quillfeldt, 2002, 2011). Some extraordinary events have been recorded, such as breeding in tree holes (Lopez et al., 2018) or quarries (Tella et al., 2014).

Besides, during several expeditions to Neotropical countries performed from 2012 to 2019, we estimated parrot abundances in the field using road surveys, stopping each time a flock was detected to identify and count individuals and observe their feeding behavior (Tella et al., 2021). This methodology, together with foot transects for detecting foraging flocks (Hernández‐Brito, Martina Carrete, et al., 2021; Hernández‐Brito, Romero‐Vidal, et al., 2021; Sebastián‐González et al., 2019), allowed us to find parrots breeding in palm tree bracts. Individuals of different secondary cavity‐nesting species (red‐shouldered Macaw, *Diopsittaca cumanensis*; yellow‐crowned amazon, *Amazona ochrocephala*; orange‐winged amazon, *Amazona amazonica*; red‐lored parrot, *Amazona autumnalis*; chesnut‐fronted macaw, *Ara severus*; finsch's parakeet, *Psittacara finschi*; scarlet‐fronted parakeet, *Psittacara wagleri*; and white‐eyed parakeet, *Psittacara leucophtalmus*) were seen excavating palm tree bracts to create cavities, with clear reproductive behavior such as bringing food to the nest while the other bird was inside the cavity. In a few cases, it was even possible to verify the presence of eggs in the nests. There are no records of breeding attempts on different substrates for any of these species.

## 3 RESULTS

**FIGURE 2:**
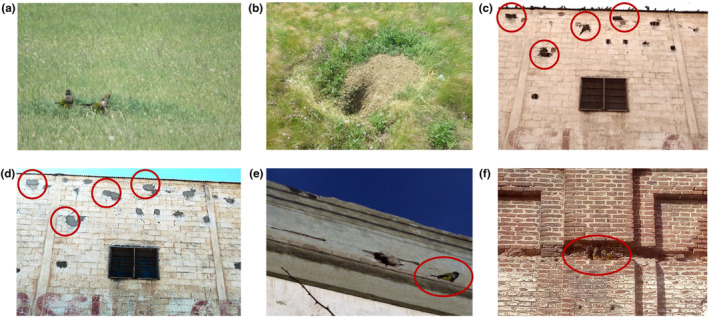
(a) Pair of burrowing parrots (*Cyanoliseus patagonus*) at the entrance of a burrowing owl (*Athene cunicularia*) nest located in the ground (b) abandoned burrow of burrowing owl, which burrowing parrots were occupying (November 2017). (c) Four pairs of burrowing parrots (*Cyanoliseus patagonus*) using holes (marked with red circles) that had previously been enlarged in the exterior walls of a building in the village of Hilario Ascasubi, Buenos Aires, Argentina (June 2018). (d) The same wall after covering the cavities with cement (February 2019). (e) Individual of the breeding pair next to the hole in Zapala building (indicated with the red circle). (f) Pair of burrowing parrots using holes in a wall of a railway warehouse in Bahía Blanca, Buenos Aires, Argentina (October 2020). Photographs (a) and (b) Álvaro Luna and Pedro Romero‐Vidal; (c) and (d) Daiana Lera and Sergio Zalba; (e) Fernando Ramírez; (f) Lucas Vernier.

During November 2017, we recorded the first evidence of burrowing parrots attempting to breed in a burrowing owl nest located in a sand dune area with human‐transformed grasslands (38°44′30″ S, 62°45′11″ W, Figures 1 and 2a,b). The presence of parrots was confirmed on subsequent visits in December, but on the last visit (January 30, 2018), there were neither parrots nor burrowing owl or other species occupying the nest. The attempt to breed in ground burrows was never recorded before for burrowing parrots.

During the 2018–2019 reproductive season, we recorded two cases of burrowing parrots nesting in holes in the walls of buildings in two small villages (Hilario Ascasubi, Buenos Aires province, 39°22′00″ S, 62°38′00″ W, and Zapala, Neuquén province, 38°54′10″ S, 70°03′54″ W; Figure 1a letter H; Figure 2c–f). In both cases, we observed numerous pairs of the species enlarging small hollows located more than three meters above ground level, on exterior walls. In one of these colonies (Hilario Ascasubi), the nesting attempt ceased when the building owners covered the holes with cement (Figure 2c,d). Although the parrots remained at the site after repeated visits, we do not know if they were successful in the breeding attempt. Similarly, a breeding pair of burrowing parrot was observed in October 2020 in Bahía Blanca (Buenos Aires province, 38°43′27.6″ S, 62°17′03.3″ W; Figure 1a letter G; Figure 2f) attempting to breed in a hole in the wall of a railway warehouse. Although the pair was observed in the same place repeatedly during 2021–2022, we could not confirm breeding success in these years.

In 2014, we observed a breeding pair of white‐eyed parakeets nesting in the bracts of a palm tree species, *Attalea speciosa* (Table 1), in a savanna‐like forest used for livestock grazing, 30 km from the city of Trinidad (Beni, Bolivia, 14°45′43″ S, 64°53′22″ W; Figure 1a letter F), representing the first observation of this nesting innovation. Parakeets enlarged the natural cavity between bracts by digging so that it resembled a cavity in a tree. However, throughout the same year and in a second expedition in 2017, we observed more breeding events in areas close to the first record site (Figure 1a, letter D), accounting for a total of 93 breeding pairs belonging to five species of parrots (Table 1) in areas with similar characteristics (Figure 1a letter E), all placed in palms of the genus *Attalea* (Table 1).

**TABLE 1:** Number of breeding pairs of each parrot species recorded nesting in palm tree bracts in the different areas. Palm tree species where nest were located are also provided.

Species	Breeding pairs	Palm tree species
**Bolivia 2014**		
*Psittacara leucophtalmus*	1	*Attalea speciosa*
*Psittacara leucophtalmus*	2	*Attalea totai*
*Diopsittaca cumanensis*	6	*Attalea totai*
*Amazona ochrocephala*	1	*Attalea totai*
**Bolivia 2017**		
*Diopsittaca cumanensis*	77	*Attalea speciosa*
*Ara severus*	4	*Attalea speciosa*
*Amazona amazonica*	2	*Attalea speciosa*
*Amazona ochrocephala*	5	*Attalea speciosa*
*Psittacara leucophtalmus*	1	*Attalea speciosa*
**Costa Rica**		
*Amazona autumnalis*	15	*Attalea rostrata*
*Psittacara finschi*	10	*Attalea rostrata*
**Panama**		
*Amazona autumnalis*	6	*Attalea butyracea*
**Colombia**		
*Psittacara wagleri*	3	*Attalea butyracea*
**Argentina**		
*Cyanoliseus patagonus*	4	*Phoenix canariensis*

**FIGURE 3:**
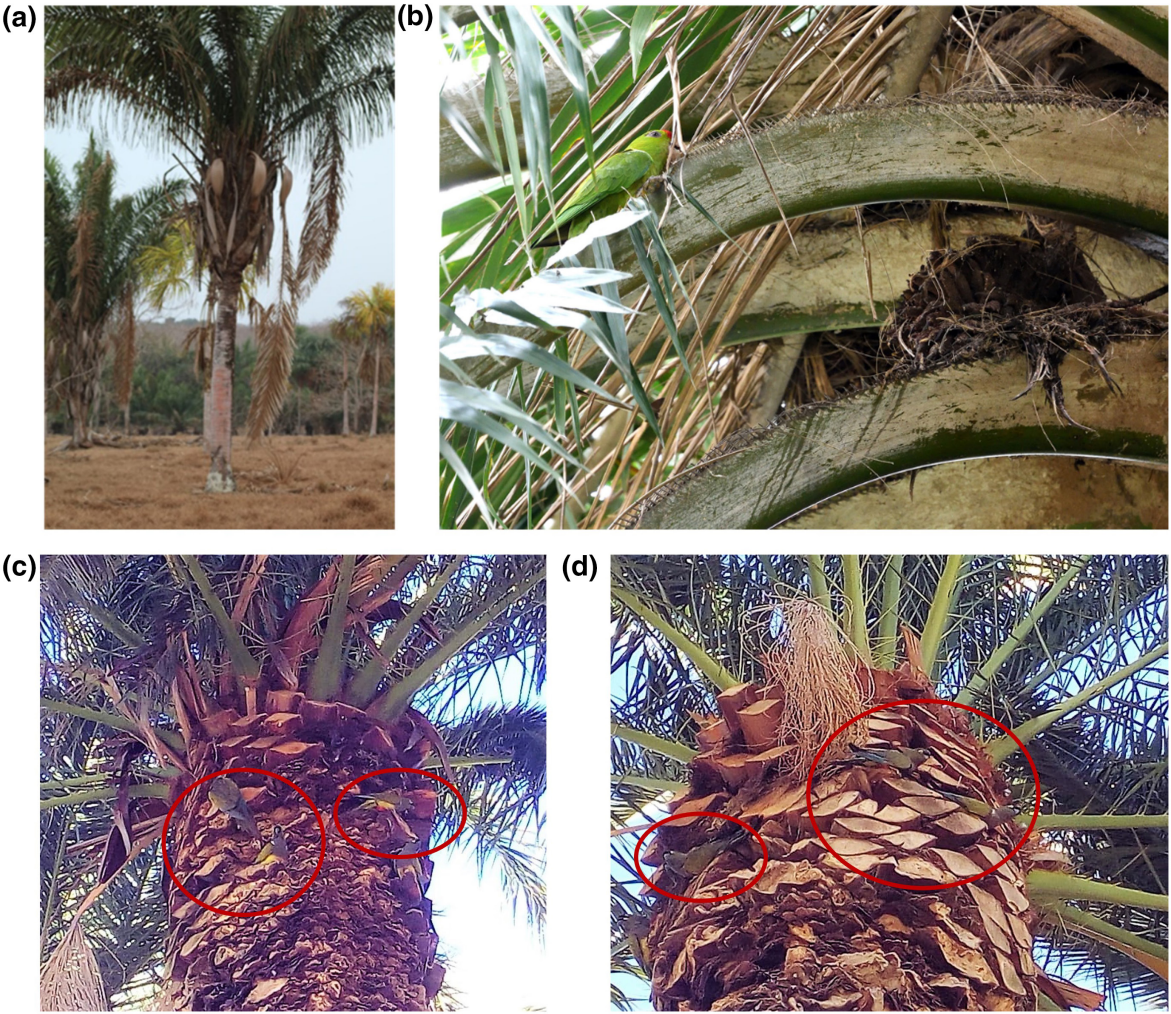
(a) *Attalea butyracea* palm tree in Darien (Panamá) in a savanna‐like area used for livestock grazing where red‐lored amazons, *Amazona autumnalis*, were observed breeding. (b) Scarlet‐fronted parakeet, *Psittacara wagleri*, breeding in the bracts of an *Attalea butyracea* in Colombia. (c) Two breeding pairs of burrowing parrots (indicated by the red circles) attempting to breed in palm tree bracts of *Phoenix canariensis* in a green area of the city of Bahía Blanca, Buenos Aires, Argentina (June 2021). (d) Two breeding pairs of burrowing parrots attempting to breed in palm tree bracts of *Phoenix canariensis* on another green area of Bahía Blanca (July 2021). Photographs: (a) Pedro Romero‐Vidal; (b) José L. Tella; (c) and (d) Lucas Vernier.

During subsequent expeditions carried out in Costa Rica in 2018 (8°42′45″ N, 83°38′42″ W; Figure 1a letter A) and Panama in 2019 (8°40′05″ N, 78°10′04″ W; Figure 1a letter B), we recorded similar nesting events, involving 31 breeding pairs of two different species (Table 1). In both cases, the habitat was similar to the Bolivian observations (Figure 3a). In addition, during an expedition to Colombia in 2019 (5°44′42″ N, 75°36′36″ W; Figure 1a letter C), three pairs of scarlet‐fronted parakeets were recorded breeding in the bracts of *Attalea butyracea* in a garden next to a restaurant where palm trees were scattered forming an open area (Figure 3b).

In 2021, we recorded four breeding pairs of burrowing parrots attempting to breed in palm tree bracts of *Phoenix canariensis* (Table 1; Figure 3c,d) in two different green areas of Bahía Blanca (Buenos Aires province, Argentina, 38°43′27.6″ S, 62°17′03.3″ W; Figure 1a letter G).

## 4 DISCUSSION

Our observations on the Neotropics of parrot species known to nest in cavities (in trees or cliffs) throughout their native ranges (Cornelius et al., 2008; Forshaw, 2010), together with data reported elsewhere (Hernández‐Brito, Martina Carrete, et al., 2021; Hernández‐Brito, Romero‐Vidal, et al., 2021; Lopez et al., 2018), suggest that the ability of these birds to use novel nesting sites is not merely anecdotal but rather an overlooked phenomenon. Factors promoting the emergence of these innovations are unknown, but the lack of suitable cavities resulting from increasing anthropogenic changes in natural habitats may be an important driver encouraging individuals to try alternative strategies. In our case, novel nests were observed in areas where the forest has been cleared for cattle grazing, leaving only palm trees probably for human exploitation (Araújo & Lopes, 2012). Despite the existence of forest patches in the surroundings, many of these areas have suffered past deforestation (Redo et al., 2012) and lack large mature trees, which provide the best nesting cavities for these species. In these cases, when food is abundant and nesting substrates are scarce, individuals may breeding in aggregations (pseudo‐colonies) in cavities excavated in palm tree bracts (Hernández‐Brito, Martina Carrete, et al., 2021; Hernández‐Brito, Romero‐Vidal, et al., 2021). It is worth mentioning that this anthropogenic habitat could also allow parrots to detect potential predators in advance (Ontiveros et al., 2005), constituting an example of enemy‐release behavior (Schlaepfer et al., 2002). However, this could also constitute an ecological trap, as breeding pairs nesting in these areas are more exposed to human threats, such as poaching, especially considering the high demand for some species such as Amazons on the illegal trade (Romero‐Vidal et al., 2020).

In the case of burrowing parrots, although rare, this species can show plasticity in its selection of nesting sites, and sometimes breeds in human structures that resemble natural substrates (Masello & Quillfeldt, 2005; Tella et al., 2014) or even in trees holes (Lopez et al., 2018). Thus, the choice of palm bracts for nesting is similar to breeding in tree holes (Lopez et al., 2018), as other parrot species do in other areas. In fact, the large number of observations involving different species at different sites suggests that this behavior may not be merely anecdotal but rather an overlooked phenomenon. On the other hand, the use of a ground nest previously excavated by burrowing owls seems to have been a poor choice, possibly due to the high risk of predation, aggravated by the lack of conspecifics. Nesting attempts on the wall of buildings by several pairs of burrowing parrots constitute an adaptation to a completely new environment. In the case of Hilario Ascasubi, the breeding pairs could have been successful if not for the closure of the holes, thus benefiting from this artificial substrate with characteristics very similar to those present in their usual nesting areas (Masello et al., 2001, 2006; Masello & Quillfeldt, 2002, 2011). Moreover, burrowing parrots would have also taken advantage of this substrate in terms of predator detection and defense by breeding colonially (Masello & Quillfeldt, 2002). In the other two cases, although it was not possible to determine the fate of the breeding attempts, it was confirmed that the breeding pairs remained in the area during the season. The use of building cavities instead of natural ones has been also observed in other parrot species, as the ring‐necked parakeets in their invasion areas (Hernández‐Brito, 2014). Nesting in buildings or other human‐made structures constitutes the best representation of nesting site innovation, as implies the adaptation to a new environment (urban) and to new substrates. Therefore, these attempts at innovation, which also involve dealing with the stress generated by humans, could be initiated by bold individuals, as in the case of urban colonization by other species (Carrete & Tella, 2013). The use of tree cavities (Lopez et al., 2018), palm tree bracts or abandoned burrows from burrowing owls may not be novel but occur at a very low frequency. However, the use of these substrates may now be increasing, together with the use of buildings, likely due to the human‐induced habitat transformations and/or to seek the proximity of humans for the new food resources they provide (i.e., exotic plant species and crops; Blanco et al., 2022).

Although the mechanisms that facilitate nest site innovations are not yet fully understood, our observations support the idea that parrots are a group of birds with a great capacity for behavioral innovation. In a scenario of global change, where important resources for wildlife will continue to be transformed, it is important not only to record these events but also to investigate the demographic consequences they have for wild populations, some of which are of conservation concern.

## AUTHOR CONTRIBUTIONS

**Pedro Romero‐Vidal:** Conceptualization (equal); data curation (lead); formal analysis (lead); investigation (equal); methodology (equal); software (lead); visualization (lead); writing – original draft (lead); writing – review and editing (equal). **Guillermo Blanco:** Conceptualization (equal); data curation (supporting); investigation (equal); methodology (equal); visualization (equal); writing – review and editing (equal). **Fernando Hiraldo:** Conceptualization (equal); investigation (equal); visualization (equal); writing – review and editing (equal). **José A. Díaz‐Luque:** Investigation (supporting); methodology (supporting); writing – review and editing (equal). **Álvaro Luna:** Conceptualization (equal); investigation (equal); methodology (equal); visualization (equal); writing – review and editing (equal). **Daiana Lera:** Investigation (equal); methodology (supporting); writing – review and editing (equal). **Sergio Zalba:** Investigation (equal); writing – review and editing (equal). **Martina Carrete:** Conceptualization (equal); funding acquisition (lead); investigation (equal); methodology (equal); project administration (lead); supervision (lead); writing – review and editing (equal). **José L. Tella:** Conceptualization (equal); funding acquisition (lead); investigation (equal); project administration (equal); supervision (lead); visualization (equal); writing – review and editing (equal).

## FUNDING INFORMATION

This work was supported by Loro Parque Fundacion (Project SEJI/2018/024) and by project CGL2015‐71378‐P from Ministerio de Economía, Industria y Competitividad (Spain). Álvaro Luna was supported by La Caixa‐Severo Ochoa International PhD Program 2015 and the Severo Ochoa Program for Centres of Excellence in R + D + I (SEV‐2012‐0262).

## CONFLICT OF INTEREST STATEMENT

The authors declare no conflict of interest.

## Data Availability

The data that supports the findings of this study are available in the tables and results section included in the article.
